# Phenotypic Responses of a Stoloniferous Clonal Plant *Buchloe dactyloides* to Scale-Dependent Nutrient Heterogeneity

**DOI:** 10.1371/journal.pone.0067396

**Published:** 2013-06-27

**Authors:** Dong Luo, Yong-Qiang Qian, Lei Han, Jun-Xiang Liu, Zhen-Yuan Sun

**Affiliations:** 1 Department of Ornamental Horticulture, College of Agriculture and Biotechnology, China Agricultural University, Beijing, China; 2 State Key Laboratory of Tree Genetics and Breeding, Research Institute of Forestry, Chinese Academy of Forestry, Beijing, China; Instituto de Biología Molecular y Celular de Plantas, Spain

## Abstract

Clonal plants could modify phenotypic responses to nutrients heterogeneously distributed both in space and time by physiological integration. It will take times to do phenotypic responses to modifications which are various in different growth periods. An optimal phenotype is reached when there is a match between nutrient conditions and foraging ability. A single plantlet of *Buchloe dactyloides* with two stolons was transplanted into heterogeneous nutrient conditions. One stolon grew in homogeneous nutrient patch, while the other cultured in different scales of heterogeneous nutrient patches. As compared to the other nutrient treatment, heterogeneous nutrient treatments with small scale of 25×25 cm resulted in a higher biomass, and larger number of ramets, clumps and stolons in *B. dactyloides* at both genet and clonal fragment levels. Significant differences of number of ramets, clumps and stolons were detected at the rapid growth stage, but not in the early stage of the experiment. Foraging ability was more efficient in heterogeneous than in homogeneous nutrient conditions as assessed by higher root mass and root to shoot ratio. Different nutrient treatments did not prompt significant differences in internode and root length. Physiological integration significantly increased biomass, but did not influence other growth or morphological characters. These results suggest that physiological integration modifies phenotypic plasticity of *B. dactyloides* for efficient foraging of nutrients in heterogeneous nutrient conditions. These effects are more pronounced at genet and clonal fragment levels when the patch scale is 25×25 cm. Time is a key factor when phenotypic plasticity of *B. dactyloides* in heterogeneous nutrient conditions is examined.

## Introduction

The availability of nutrients in natural habitats occurs at a variety of spatial and temporal scales. De Kroon *et al.* [1] proposed that a whole plant consists of many modules. Clonal plants can form large interconnected systems consisting of numerous connected modules for a period of time via the clonal growth [2]–[4]. Thus, the modules of a single clonal plant may occupy different quality patches [5]–[11]. This results in different parts of the same module or different modules of the same plant, expressing a range of phenotypic responses to their local conditions [12], [13].

Physiological integration is one of the most important traits of clonal plants as it can optimize the resource allocation among modules of the whole plant. It also allows transporting photosynthates, water, nutrients, and signals via connected stolon or rhizome internodes from source modules to sink modules of the same genet [14], [15]. Physiological integration increases the capacity of resource acquisition and utilization for all integrated modules, and helps clonal plants to cope with complex habitats [16]–[27]. Since it will take times to do morphological reactions to physiological responses which are various in different growth periods, clonal plants may employ phenotypic plasticity as a behavioral adaptation to modify the suitability of variable habitats, exploiting favorable and avoiding unfavorable patches of habitats, by changing the responses at different growth periods [28]–[33]. De Kroon and Hutchings [34] indicated that increased branching in favorable conditions could be interpreted as a positive growth response. Plants might shorten their internodes to form the clumping ramets in order to maximize the acquisition of required nutrients in a local environment under favorable conditions. On the other hand, these internodes were extended to form the spreading ramets that explore new habitats when they grew in unfavorable conditions. Physiological integration among ramets modified morphological, physiological and mycorrhizal plasticity of roots to allow locating more roots in favorable patches to maximize the nutrient acquisition from heterogeneous habitats [35]–[40].

Phenotypic responses of clonal plants to different nutrients depend on the spatial and temporal distribution of the nutrient, the size of patches selected for ramet placement, and the ability of nutrient acquisition [41]–[43]. Positive phenotypic responses are shown when all the above described factors are in harmony. Reactions of clonal plants to habitats involve the responses of its modules to localized conditions, and these responses are modified through physiological integration with other modules exposed to different conditions. This results in an integrated and adaptive response at the levels of individual modules and the whole plant. The present study was conducted to test the phenotypic responses of *B. dactyloides* to heterogeneous nutrients of different scales, measured as growth and morphological characters in homogeneous and three scales of heterogeneous treatments with the same amounts of essential nutrients in different growth periods. Phenotypic plasticity of a clonal plant under heterogeneous environments is affected by local conditions as well as the interactions between connected modules of the same genet in contrasting conditions [1]. We thus also attempted to test the effects of physiological integration among the clonal fragments of *B. dactyloides* in heterogeneous nutrient conditions on phenotypic responses.

## Materials and Methods

### Plant Species and Experimental Material

*Buchloe dactyloides*, buffalograss, is a perennial herb of the Poaceae family with ramets connected by aboveground stolons. It can form large, morphologically and physically interconnected systems consisting of clonal modules of different levels via clonal growth [44]. The plant material used in this study was a *B. dactyloides* clonal ramet with two new connected stolons, and the ramets were all in the same genotype and similar in size.

### Experimental Design

**Figure 1:**
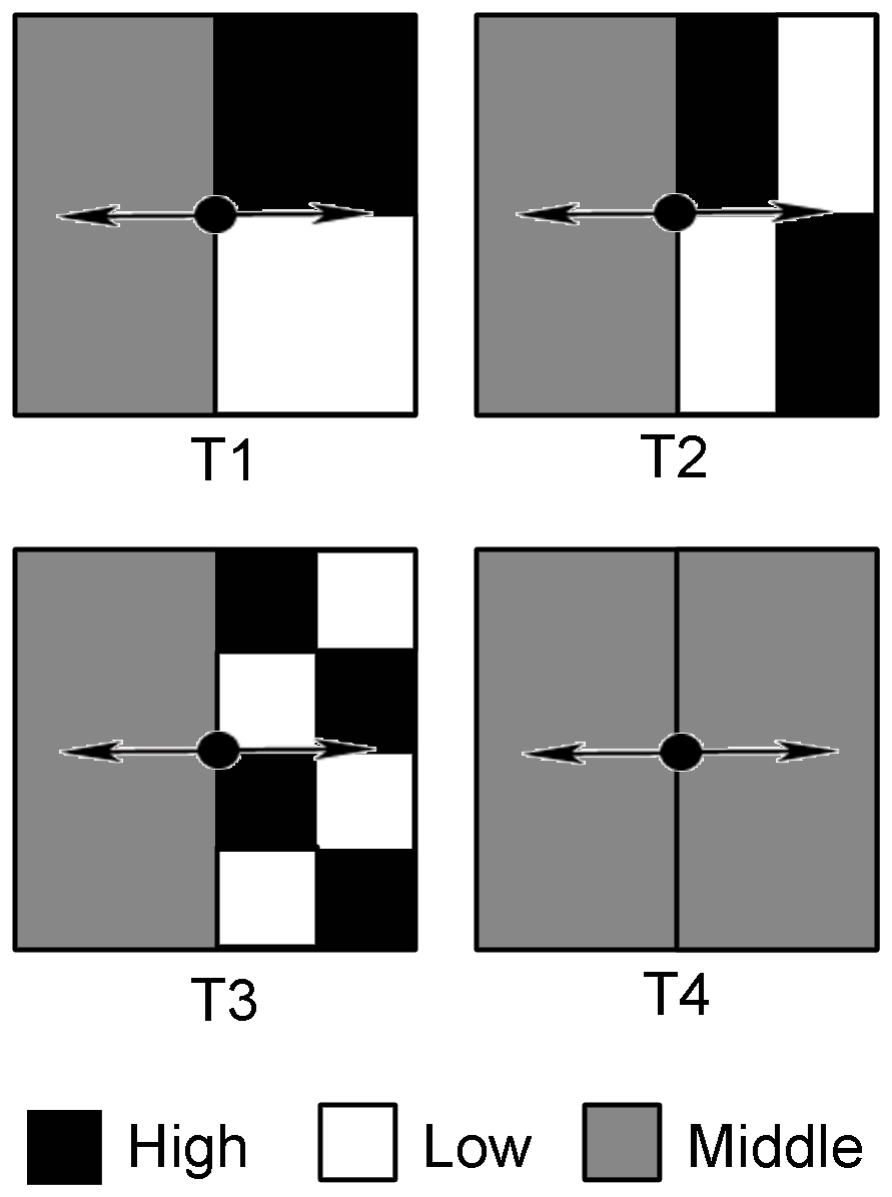
Experimental design. The boxex used in this study were 100×100×25 cm in size. Three scales of heterogeneous nutrient treatments and one homogeneous nutrient treatment, coded as T1, T2, T3 and T4, were implemented in the boxes. The box was divided into two parts, one was filled with homogeneous nutrient and the other with 3 scales of heterogeneous nutrients (the three small boxes were divided respectively into two, four and eight patches, half of patches in each small box were filled with high nutrient and the other half low nutrient).

The experiment was carried out in natural light conditions at the Nan Yuan field station of Chinese Academy of Forestry (CAF) in early 2012 during the growing season. The boxes with dimensions 100×100×25 cm in size were placed into the field and filled with immature soil as the substrate. Four different nutrient treatments including one homogeneous nutrient and three scales of heterogeneous nutrient treatments were given within the boxes (Fig. 1). Treatment 1 (T1) consisted of one homogeneous and one large scale (50×50 cm) heterogeneous nutrient patches; Treatment 2 (T2) consisted of one homogeneous and one middle scale (50×25 cm) heterogeneous nutrient patches; Treatment 3 (T3) consisted of one homogeneous and one small scale (25×25 cm) heterogeneous nutrient patches; Treatment 4 (T4) consisted of two homogeneous nutrient patches. Adjacent nutrients in the same box were physically separated and a single plantlet with two stolons was transplanted to the centre of the box. It might acquire additional resources when the ramet extends over the box. This will affect results of experiment. Thus, the plants were allowed to grow for only seven weeks to ensure not rooting beyond the boxes. There were six replicates for each nutrient treatment. The total nutrient supply was the same in all nutrient treatments. Each treatment contained 150.0 g of controlled release fertilizer Osmocote *313s* (16-9-12-2.5MGO +TE, Everris, ICL Group). This nutrient was considered sufficient maintaining the growth of *B. dactyloide* during the experiment.

### Harvests and Measurements

The growth characters included the number of ramets, clumps and stolons, aboveground mass, root mass, total mass and root to shoot ratio. The clumps were defined as the nodes from which the new ramets, stolons and roots produced. The morphological characters included the internode and root length. The number of ramets, clumps and stolons were recorded once a week. After seven weeks of growth, the aboveground and root parts of *B. dactyloides* were harvested separately, dried at 108°C for 30 min, then at 80°C to a constant weight, and biomass was recorded. The morphological characters were also measured. All the characters were recorded separately from each nutrient patch.

### Data Analysis

Multivariate analysis of variance (MANOVA) followed by Duncan’s Multiple Range Tests (DMRT) was used to compare the differences of number of ramets, number of clumps and number of stolons surveyed at the same time among the four different treatments at genet level, and between homogeneous and heterogeneous patches of the same treatment at clonal fragment level. Independent *t*-test was used to determine the means of the growth and morphological characters between homogeneous and heterogeneous patches of the same treatment at the end of the experiment (on the 49^th^ day). One-way ANOVA followed by DMRT was used to compare the means of growth and morphological characters among homogeneous patch of homogeneous nutrient treatment and heterogeneous patches of the three scales of heterogeneous nutrient treatments on the 49^th^ day. Analyses in this study were conducted with SPSS 16.0 (SPSS, Chicago, IL, USA).

## Results

**Figure 2:**
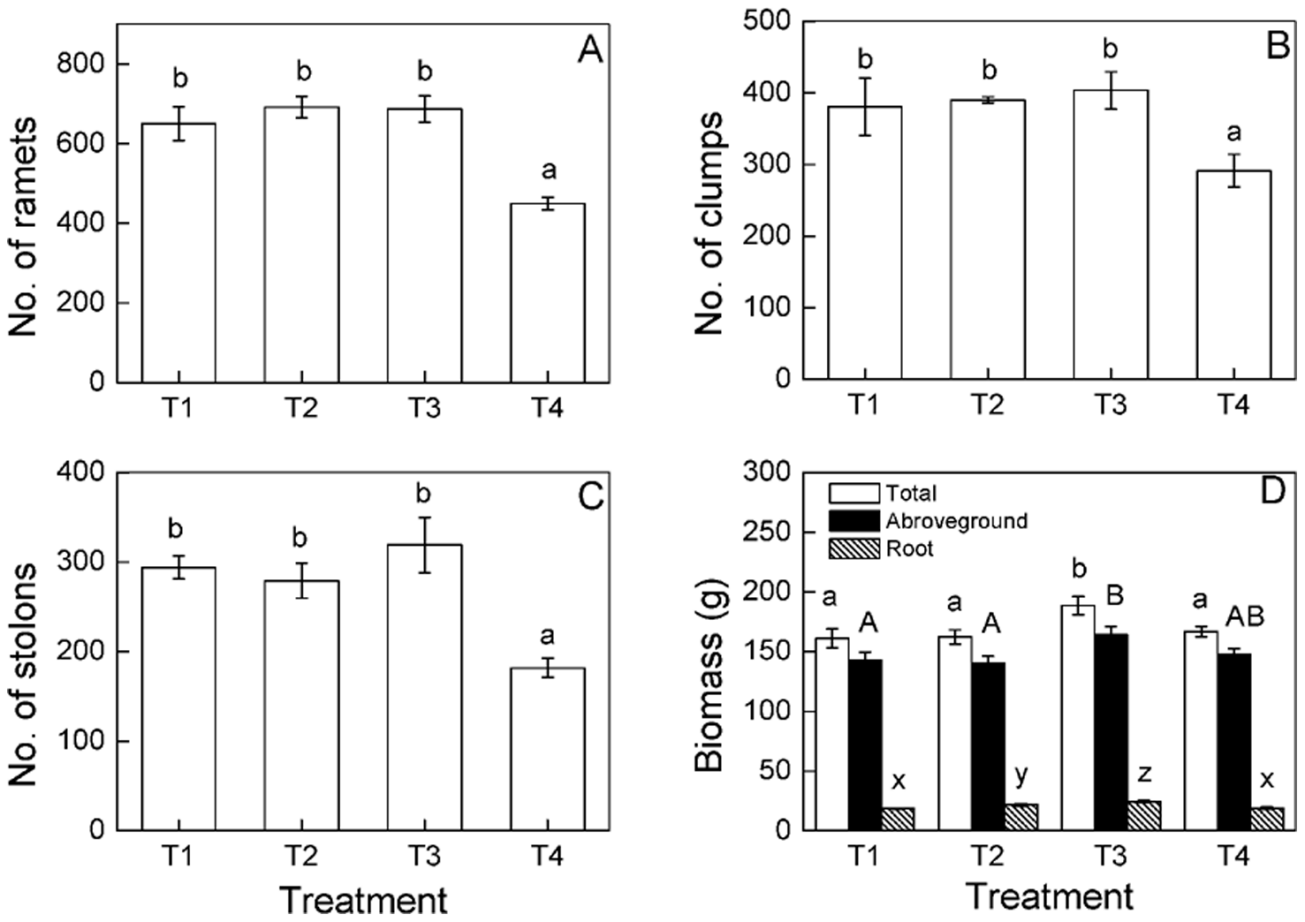
Responses of growth characters of *Buchloe dactyloides* to heterogeneous nutrients at genet level. The growth characters include number of ramets (A), number of clumps (B), number of stolons (C) and biomass (D). The biomass include total mass, aboveground mass and root mass. Bars are mean values (± S. E.). Bars sharing the same letters are not different at *p* = 0.05. Treatment codes are in Figure 1.

**Figure 3:**
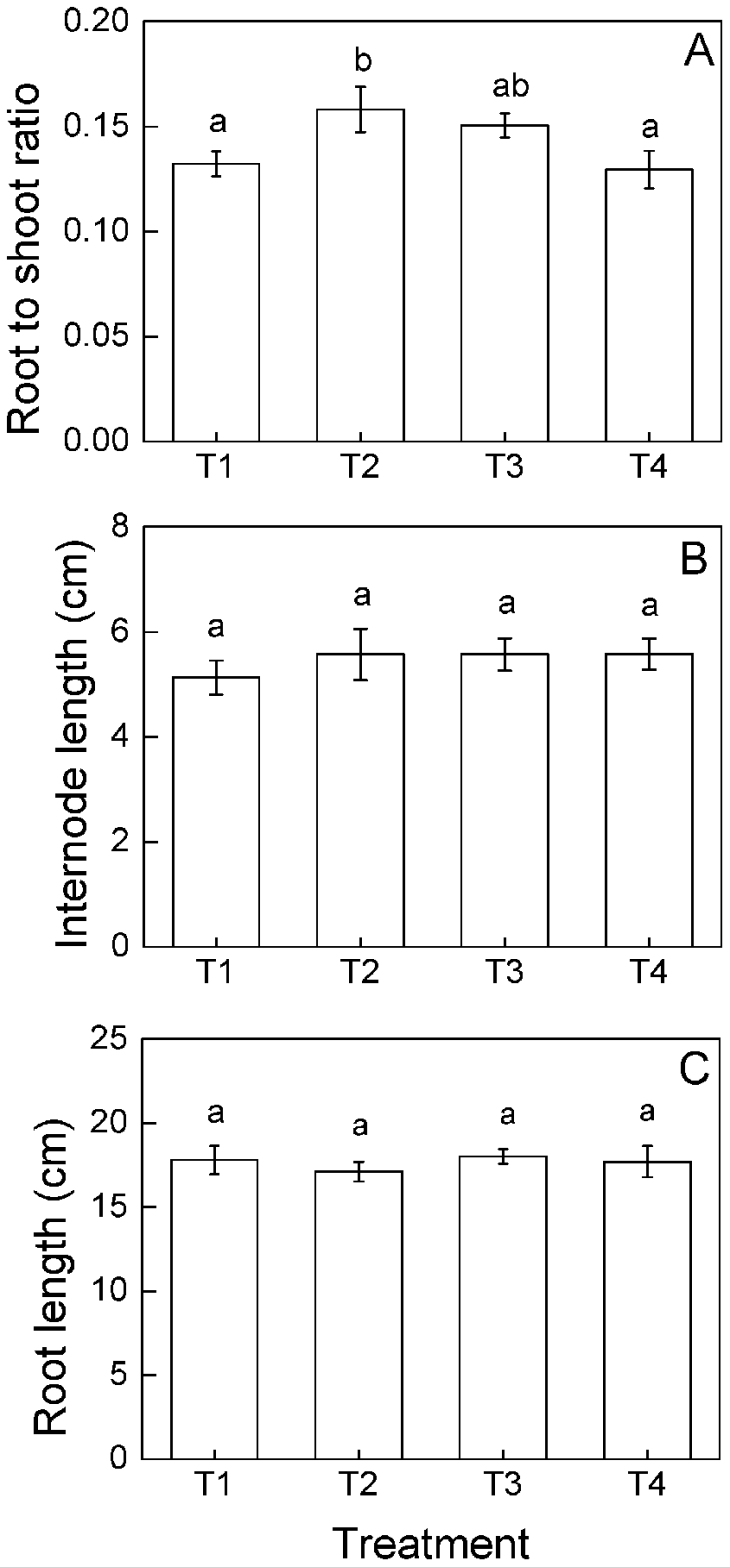
Responses of root to shoot ratio and morphological characters of *Buchloe dactyloides* to heterogeneous nutrients at genet level. Mean values (± S. E.) of root to shoot ratio (A), internode length (B) and root length (C) are given. Bars sharing the same letters are not different at *p* = 0.05. Treatment codes are in Figure 1.

The number of ramets, clumps and stolons significantly increase as time progressed, and the significant differences were found among the four nutrient treatments at different times (Fig. 2, Table 1), the number of ramets and clumps differed on the 7^th^, 14^th^, 42^nd^ and 49^th^ days while the number of stolons differed on the 14^th^, 35^th^ and 49^th^ days (Table 2). At the end of the experiment (the 49^th^ day), the number of ramets, clumps and stolons were higher in the heterogeneous (T1, T2 and T3) than in the homogeneous nutrient treatments (T4), but no significant differences were observed among the three heterogeneous nutrient treatments (Fig. 2A, B, C). Aboveground mass, root mass and total mass were the highest at the small scale (T3, 25×25 cm) heterogeneous nutrient treatment (Fig. 2D). Root to shoot ratio was the highest at the middle scale (T2, 50×25 cm) heterogeneous nutrient treatment (Fig. 3A). No significant differences were observed in internode length or root length among the four nutrient treatments (Fig. 3B, C).

**Table 1:** Analysis of variance results of *Buchloe dactyloides* growth characters in different nutrient treatments on different days at genet level.

	Time (d)						
Character	7	14	21	28	35	42	49
No. of ramets	3.72*	7.61**	1.82^ns^	1.25^ns^	1.47^ns^	3.81*	3.72*
No. of clumps	5.70**	6.37**	2.01^ns^	1.72^ns^	1.83^ns^	5.12**	13.58***
No. of stolons	0.73^ns^	4.55*	1.39^ns^	2.92^ns^	3.43*	6.50**	9.07**

*F* values and significance levels (*** means *P<*0.001, ** means *P<*0.01, * means *P<*0.05, ns means *P*≥0.05 ).

**Table 2:** Analysis of variance results of *Buchloe dactyloides* growth characters in different nutrient treatments on different days at clonal fragment level.

		Time (d)						
Character	Treatments	7	14	21	28	35	42	49
No. of ramets	T1	0.02^ns^	0.68^ns^	0.32^ns^	1.11^ns^	0.21^ns^	0.20^ns^	2.06^ns^
	T2	0.00^ns^	0.59^ns^	0.27^ns^	0.13^ns^	0.24^ns^	13.73**	35.47***
	T3	0.00^ns^	0.03^ns^	0.09^ns^	0.74^ns^	0.69^ns^	6.18*	14.22**
	T4	0.17^ns^	0.01^ns^	0.13^ns^	0.36^ns^	0.18^ns^	0.25^ns^	0.54
No. of clumps	T1	0.29^ns^	0.59^ns^	0.61^ns^	0.07^ns^	0.12^ns^	0.85^ns^	1.82^ns^
	T2	0.00^ns^	0.63^ns^	0.41^ns^	0.15^ns^	0.46^ns^	20.32**	24.56**
	T3	0.22^ns^	0.03^ns^	0.35^ns^	0.60^ns^	0.35^ns^	7.08*	8.03*
	T4	0.17^ns^	0.04^ns^	0.00^ns^	0.40^ns^	0.44^ns^	1.14^ns^	0.18^ns^
No. of stolons	T1	–	0.33^ns^	0.81^ns^	0.27^ns^	0.04^ns^	0.17^ns^	1.04^ns^
	T2	0.09^ns^	0.02^ns^	0.42^ns^	0.03^ns^	0.00^ns^	17.37**	17.74**
	T3	–	0.00^ns^	0.37^ns^	0.47^ns^	0.39^ns^	6.97*	7.08*
	T4	0.09^ns^	0.07^ns^	0.05^ns^	0.00^ns^	0.29^ns^	0.03^ns^	0.06^ns^

*F* values and significance levels (*** means *P<*0.001, ** means *P<*0.01, * means *P<*0.05, ns means *P*≥0.05 ).

**Figure 4:**
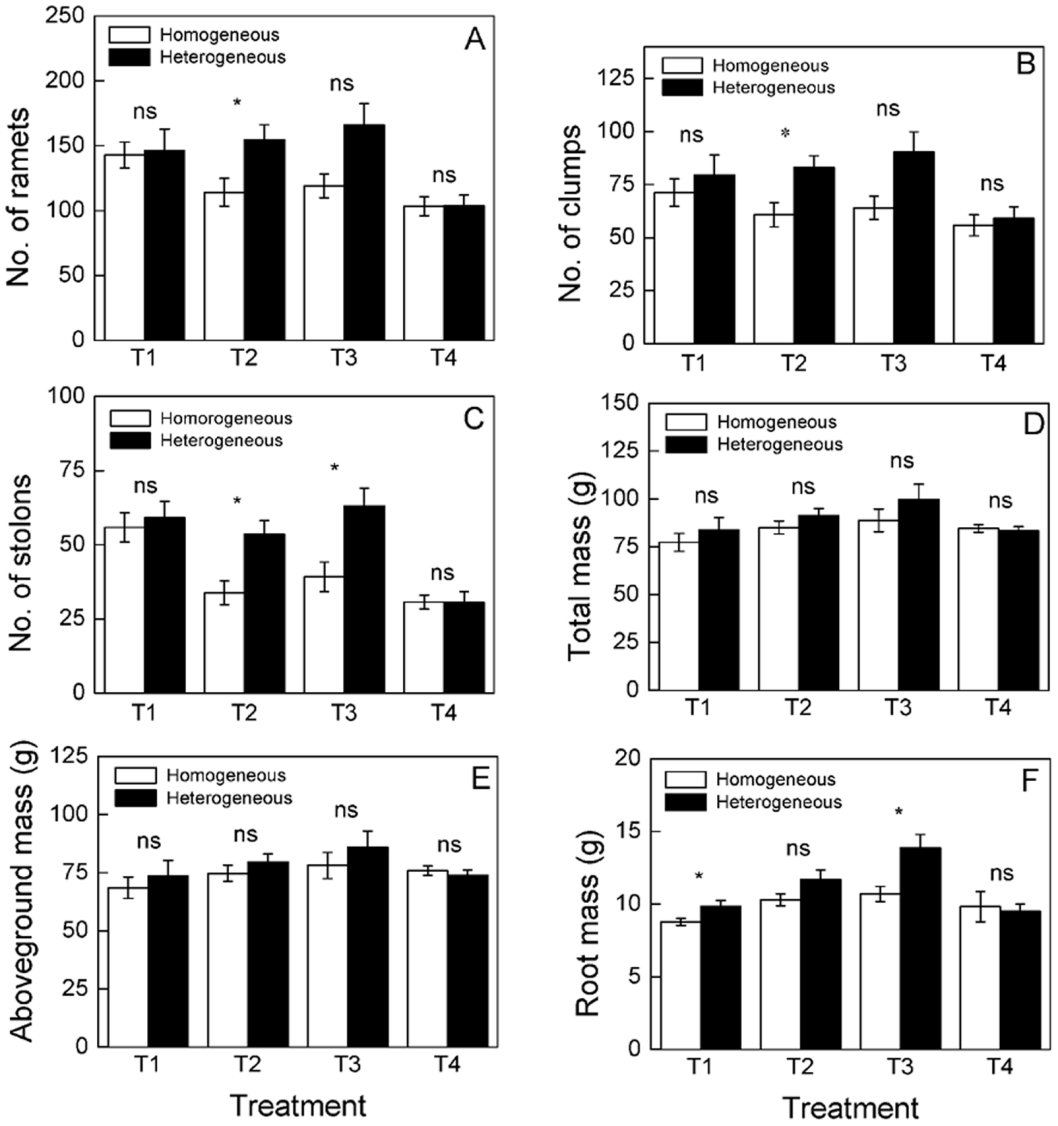
Different responses of growth characters of *Buchloe dactyloides* between homogeneous and heterogeneous patches of the same treatment at clonal fragment level. The growth characters include number of ramets, clumps, stolons, aboveground mass, root mass and total mass. Mean values (± S. E.) of number of ramets (A), number of clumps (B), number of stolons (C), total mass (D), aboveground mass (E) and root mass (F) between the homogeneous and heterogeneous patches of the same treatment are given. * means *P<*0.05. Mean values (± S. E.) of the growth characters in the heterogeneous (D) and homogeneous patches (E) are given. * means *P<*0.05. ns means *P>*0.05. Treatment codes are in Figure 1.

**Figure 5:**
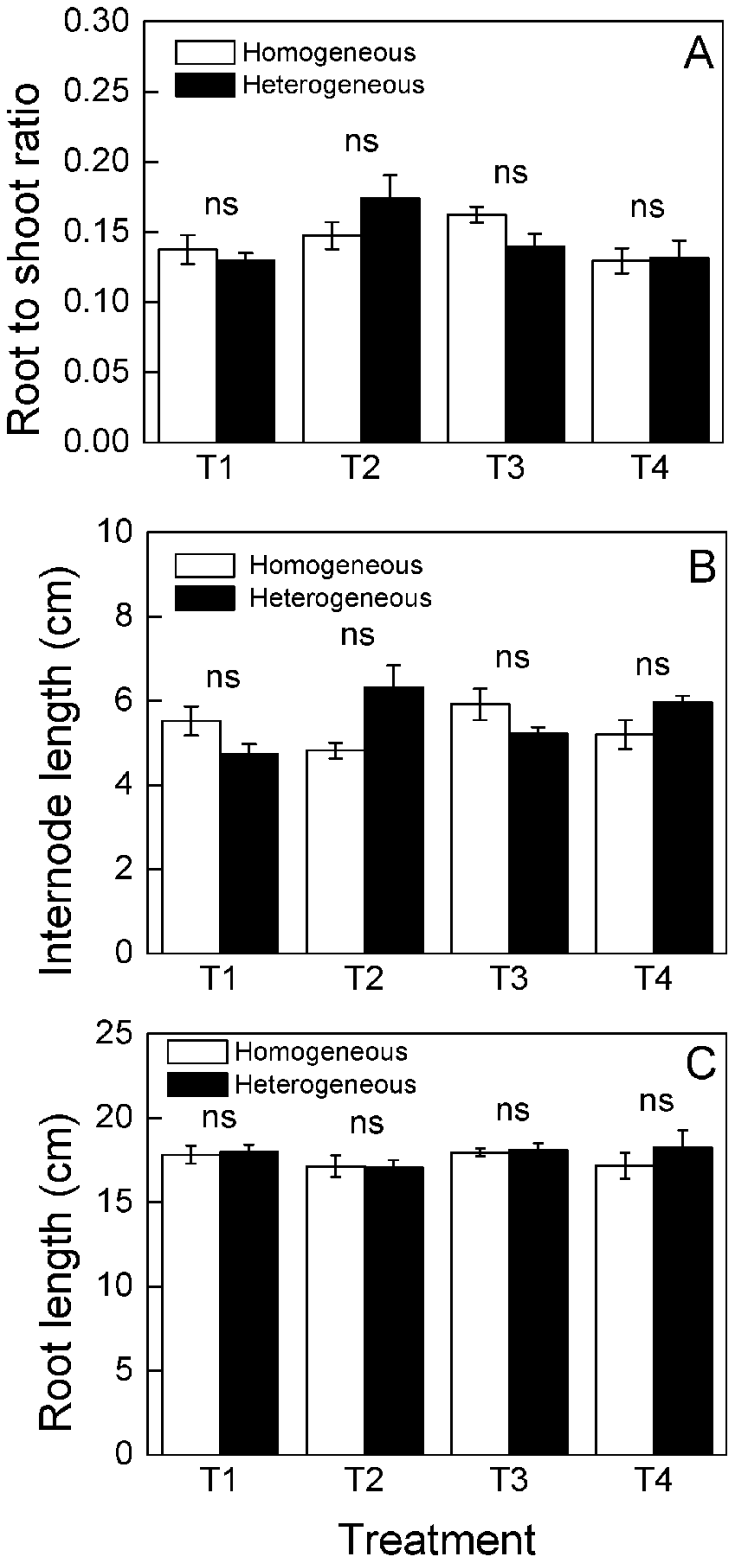
Different responses of root to shoot ratio and morphological characters of *Buchloe dactyloides* between homogeneous and heterogeneous patches at clonal fragment level. Mean values (± S. E.) of root to shoot ratio (A), internode length (B) and root length (C) between the homogeneous and heterogeneous patches of the same treatment are given. * means *P<*0.05. ns means *P>*0.05. Treatment codes are in Figure 1.

The number of ramets, clumps and stolons significantly increased as time progressed, and the significant differences were found between the homogeneous and heterogeneous patches of the same nutrient treatments at different times (Fig. 4A, B, C, Table 2), the number of ramets and clumps were higher in heterogeneous than in homogeneous patches of the same middle or small scale (T2, 50×25 cm or T3, 25×25 cm) nutrient treatment on the 42^nd^ and 49^th^ days (Table 2). Root mass was higher in heterogeneous than homogeneous patches of the same large or small scale (T1, 50×50 cm or T3, 25×25 cm) nutrient treatment (Fig. 4F), while no significant differences were observed in aboveground mass, total mass, root to shoot ratio, internode length or root length (Figs. 4D, E, 5A, B, C).

**Figure 6:**
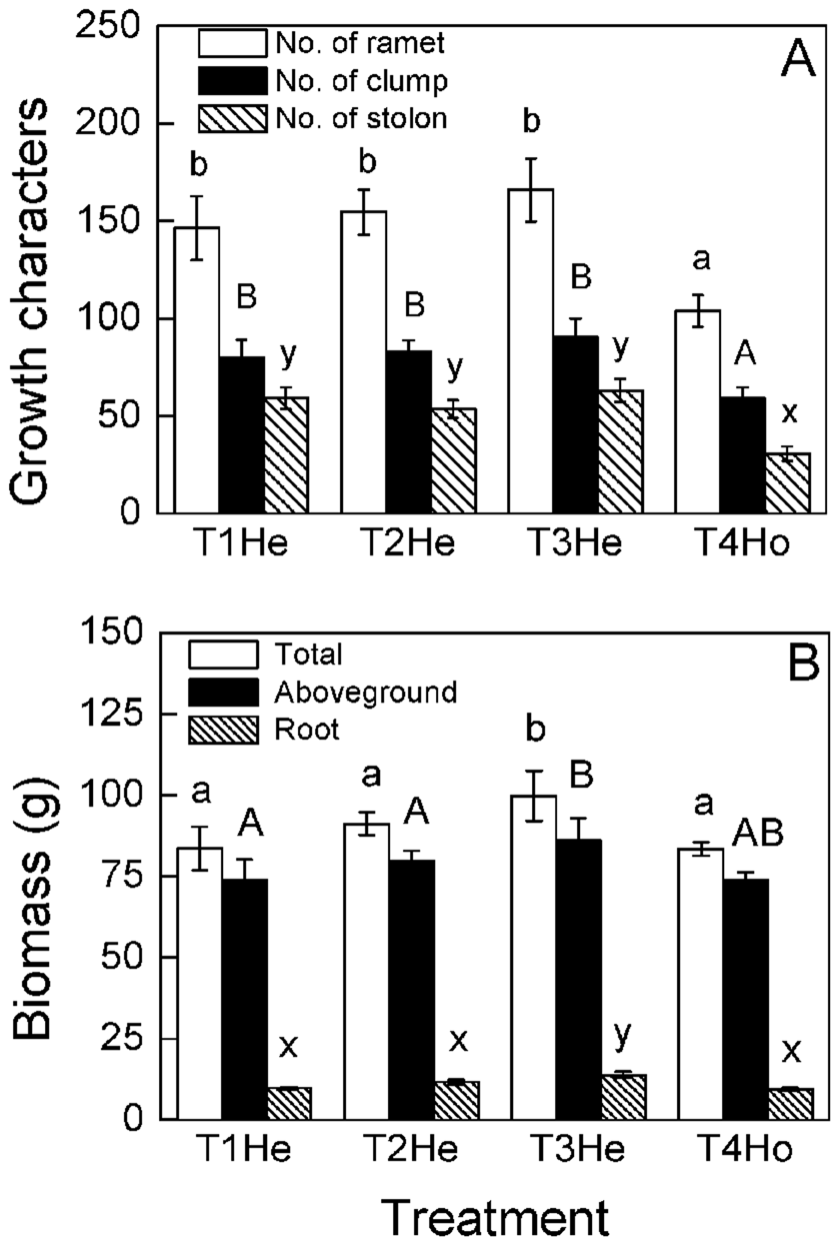
Different responses of growth characters of *Buchloe dactyloides* among heterogeneous patches of different nutrient treatments at clonal fragment level. The growth characters include number of ramets, clumps, stolons (A) and biomass (B). Bars are mean values (± S. E.). Bars sharing the same letters are not different at *p* = 0.05. Treatment codes are in Figure 1.

**Figure 7:**
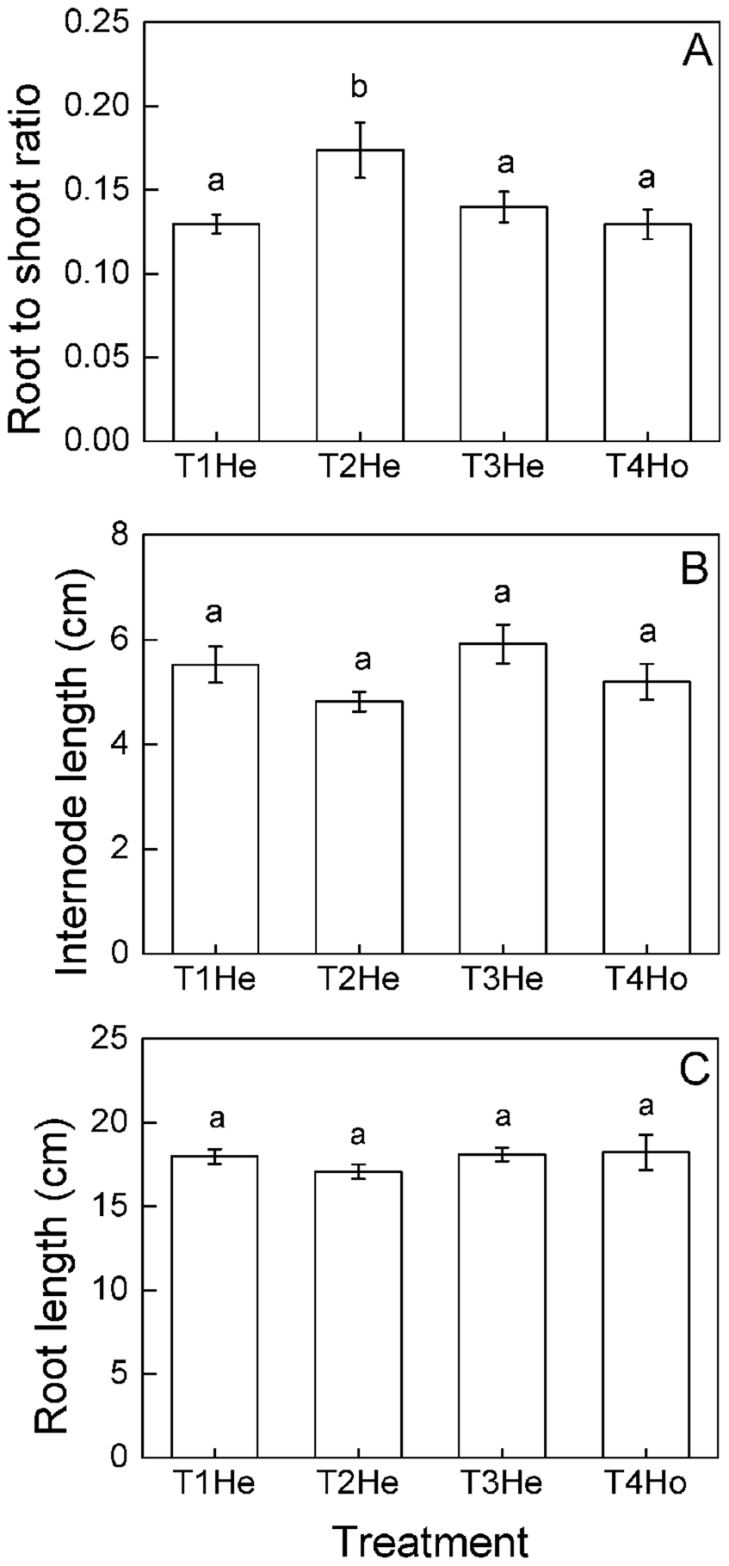
Different responses of root to shoot ratio and morphological characters of *Buchloe dactyloides* among heterogeneous patches of different nutrient treatments at clonal fragment level. Mean values (± S. E.) of root to shoot ratio (A), internode length (B) and root length (C) are given. Bars sharing the same letters are not different at *p* = 0.05. Treatment codes are in Figure 1.

The number of ramets, clumps and stolons were higher in heterogeneous patches of heterogeneous nutrient treatments than homogeneous patch of homogeneous nutrient treatment, but no significant differences were observed in heterogeneous patches of the three scales of heterogeneous nutrient treatments (Fig. 6A). Aboveground mass, root mass, total mass and root to shoot ratio were highest in small scale (T3, 25×25 cm) heterogeneous patches (Figs. 6B, 7A). No significant differences were observed in internode and root length (Fig. 7B, C). The number of ramets, clumps and stolons were larger in homogeneous patches of large scale (T1, 50×50 cm) heterogeneous nutrient treatments than the homogeneous patches of the other treatments (T2-T4, Fig. 6A). Aboveground mass, root mass and total mass were the highest in homogeneous patches of small scale (T3, 25×25 cm) heterogeneous nutrient treatments (Fig. 6B). No significant differences were found in root to shoot ratio, internode length or root length (Fig. 7A, B, C).

## Discussion

Physiological integration among clonal ramets modifies the growth of clonal plant at module and genet level, enables the plants to cope with complex habitats [45]–[55]. The growth of *B. dactyloides* was found to be significantly affected by nutrient distribution in soil. Compared to the homogeneous nutrient conditions, the growth characters were relatively higher in the heterogeneous conditions at genet level. These results are concurrent with previous reports on the effect of nutrient distribution on clonal growth [10], [56]–[58]. Significant differences were also detected in the heterogeneous nutrient conditions at clonal fragment level. Meanwhile, the phenotypic response to heterogeneous nutrients was also strongly determined by the nutrient patch scale. Since the growth characters were greater in the 25×25 cm nutrient conditions at both genet and clonal fragment levels, the phenotypic responses of *B. dactyloides* to heterogeneous nutrient conditions were more effective in small-size patch [41].

In contrast to the research of Wijesinghe and Hutchings [41], no significant differences were found in root length highlighting the physiological integration did not affect the morphological plasticity to all nutrient conditions in this study, however, the aboveground mass and root mass showed a greater root to shoot ratio. This indicates the roots to grow better in heterogeneous at the genet and clonal fragment levels especially at the smaller scale of 50×25 cm or 25×25 cm [59]–[61]. There were no marked differences in internode length in either the heterogeneous or the homogeneous nutrient conditions. This was in contrast to the escape theory that posited that ramets growing in worse conditions generated a smaller number of longer internodes to escape the unfavorable conditions [62]–[66]. Nevertheless, a greater root biomass and the same internode length could be an indication of a stronger nutrient-capture capacity in heterogeneous nutrient conditions. Therefore, our study demonstrates that the physiological integration increases the nutrient-foraging capability of *B. dactyloides* clonal ramets for acquiring more nutrients from the middle and small scales nutrient conditions.

The phenotypic responses of clonal plants are various at different growth and development stages, and the clonal plants by their own expansion might themselves reduce heterogeneity among different environments. A few studies have implicated the time as a key factor to examine their phenotypic response to heterogeneous nutrients [28]–[33]. However, at the early stage, differences due to nutrient variability were not detected in number of ramets, clumps and stolons. As a result of the preferential allocation of the ramet to different nutrient conditions, significant differences were found at the rapid growth stage, when relatively optimal ramet spacing was achieved in small scale heterogeneous nutrient conditions. Significant changes in growth characters were observed because of different growth stages but not the nutrient depletion [67], [68].

In conclusion, our results support the module concept of de Kroon [1], and indicate that physiological integration modifies the phenotypic responses of *B. dactyloides* clonal ramets for efficient foraging of nutrients in heterogeneous nutrient conditions. These effects are more pronounced at genet and clonal fragment levels in the patch scale of 25×25 cm. For the different morphological responses at different growth periods, time should be taken into account when examine the phenotypic plasticity of *B. dactyloides* in heterogeneous nutrient conditions.
